# Effect of Dexamethasone Intraligamentary Injection on Post-Endodontic Pain in Patients with Symptomatic Irreversible Pulpitis: A Randomized Controlled Clinical Trial

**DOI:** 10.22037/iej.2016.2

**Published:** 2016

**Authors:** Payman Mehrvarzfar, Ehsan Esnashari, Reyhaneh Salmanzadeh, Mahta Fazlyab, Mahyar Fazlyab

**Affiliations:** a *Department of Endodontics, Dental Branch, Islamic Azad University of Medical Sciences, Tehran, Iran;*; b *Dentist, Tehran, Iran;*; c * Iranian Center for Endodontic Research, Research Institute of Dental Sciences, Dental School, Shahid Beheshti University of Medical Sciences, Tehran, Iran; *; d *Department of Electrical and Systems Engineering, University of Pennsylvania, Philadelphia, USA*

**Keywords:** Dexamethasone, Endodontic Treatment, Intraligamentary Injection, Post endodontic Pain, Symptomatic Irreversible Pulpitis

## Abstract

**Introduction:**

The aim of this randomized-controlled clinical trial was to assess the effect of intraligamentary (PDL) injection of dexamethasone on onset and severity of post-treatment pain in patients with symptomatic irreversible pulpitis.

**Methods and Materials:**

A total number of 60 volunteers were included according to the inclusion criteria and were assigned to three groups (*n*=20). After administration of local anesthesia and before treatment, group 1 (control) PDL injection was done with syringe containing empty cartridge, while in groups 2 and 3 the PDL injection was done with 0.2 mL of 2% lidocaine or dexamethasone (8 mg/2 mL), respectively. Immediately after endodontic treatment patients were requested to mark their level of pain on a visual analogue scale (VAS) during the next 48 h (on 6, 12, 24 and 48-h intervals). They were also asked to mention whether analgesics were taken and its dosage. Considering the 0-170 markings on the VAS ruler, the level of pain was scored as follows: *score 0* (mild pain; 0-56), *score 1* (moderate pain; 57-113) and *score 3* (severe pain; 114-170). The data were analyzed using the Kruskal-Wallis and the Chi-square tests and the level of significance was set at 0.05.

**Results:**

After 6 and 12 h, group 1 and group 3 had the highest and lowest pain values, respectively (*P*<0.01 and *P*<0.001 for 6 and 12 h, respectively). However, after 24 and 48 h the difference in the pain was not significant between groups 1 and 2 (*P*<0.6) but group 3 had lower pain levels (*P*<0.01 and *P*<0.8 for 24 and 48 h, respectively).

**Conclusion:**

Pretreatment PDL injection of dexamethasone can significantly reduce the post-treatment endodontic pain in patients with symptomatic irreversible pulpitis.

## Introduction

Management of endodontic pain has a positive impact on reducing fear and anxiety of endodontic patients [1, 2]. Endodontic pain management encompasses all aspects of treatment; preoperative pain control includes accurate diagnosis and anxiety reduction; while intraoperative pain control mainly depends on effective local anesthetic/operative techniques. Comprehensive knowledge of local anesthetic solutions and their in-time proper use are necessary for pain-free treatment experience of endodontic patients [1]. However, management of postoperative pain may be as important if not superior. Most of the endodontic patients believe that pain perception subsequent to endodontic treatment of a tooth is unavoidable, yet several clinical trials indicated that postoperative pain occurs not in all endodontic patients [3, 4]. Control of post-endodontic pain can involve a variety of techniques and pharmacologic agents [1, 3].

Ineffectiveness of local anesthesia in symptomatic teeth with irreversible pulpitis has always been a matter of complexity for both patient and the clinician. Various mechanisms have been proposed to explain this phenomenon including the sensitization or activation of nociceptors by inflammation-released cytokines (such as prostaglandins) and associated central mechanisms [5, 6], *etc*. Prostaglandins which are terminal products of arachidonic acid cyclooxygenase (COX) pathway metabolism [7]; can sensitize the nociceptors to bradykinin and histamine factors as the main agents of inflammatory soup in inflamed pulps [2, 5, 7]. 

On the other hand, the endodontic treatment by itself can cause the release of inflammatory mediators (*e.g.* prostaglandins, leukotrienes, bradykinin, platelet-activating factor and substance P) into the surrounding periapical tissues [6]. As a result, pain fibers are directly stimulated (by bradykinin for instance) or sensitized (by prostaglandins) *in situ* [8]. In addition, the vascular dilation and increased permeability as a consequence of periradicular inflammation, causes edema and increased interstitial tissue response [6].

Considering the role of prostaglandins on endodontic pain, a possible strategy for reduction of post-operative endodontic pain might be the local use of an anti-inflammatory agents adjacent to the inflamed tooth to decrease the production of inflammatory mediators [1, 6] and improve the efficacy of local anesthetics. Dexamethasone is a potent glucocorticoid with anti-inflammatory efficacy 25 times more than that of hydrocortisone [1]. Mehrvarzfar *et al.* [6] have shown that supra periosteal infiltration of dexamethasone can reduce or even prevent postoperative pain in patients with irreversible pulpitis.

The purpose of this double-blind placebo controlled clinical trial was to compare the effect of intra-ligamentary injection of dexamethasone (8 mg/2 mL) and 2% lidocaine with 1:80000 epinephrine on postoperative pain of endodontic patients with symptomatic irreversible pulpitis. The null hypothesis was that there is no difference in the incidence of post-endodontic pain in patients receiving intra-ligamentary injection of dexamethasone and 2% lidocaine during endodontic treatment.

## Materials and Methods

The study protocol was evaluated and approved by Human Ethics Committee of Azad University, Dental Branch, Tehran, Iran. After performing a pilot study on 6 patients, using sample size calculation menu of Minitab, the minimum sample size for each group was estimated to be 20 (total sample size=60). 

The inclusion criteria for patient selection were as follows: age range of 18-65 years, systemically healthy patient (ASA *I* or *II*), and necessity of endodontic treatment on maxillary/mandibular first or second vital molars, clinical manifestations of symptomatic irreversible pulpitis, absence of widening in the periodontal ligament (PDL) and periapical lucency of endodontic origin on parallel periapical radiographies. Pulpal status was determined after testing with EndoIce frozen gas (Coltene/Whaledent, Inc., Mahwah, NJ, USA) and electric pulp tester (Analytic Technology, Redmond, WA, USA). An uncomfortable sensation or pain that had the tendency to linger as a dull ache after termination of the stimulus was considered abnormal [9] and a pain score of ≥56 degrees (moderate to severe) on numeric visual analogue scale (VAS) indicated by the patient were necessary inclusion criteria [10].

Likewise the exclusion criteria included: systematic complexity (ASA *III*, *IV*, and *V*), pregnancy and nursing, age less than 18 and more than 65, any contraindication or sensitivity to corticosteroids, gastrointestinal disorders, hemostatic disorders or anti-coagulant therapy during the last month, consumption of opioid or non-opioid analgesics, corticosteroids, three cyclic anti-depressants and *etc*. during the last 12 h before treatment.

Volunteers who met the criteria had to sign a fully informed consent. The demographic data of patients were meticulously recorded. The endodontic treatment started with injection of 1.8 mL of 2% lidocaine containing 1:80000 epinephrine (Darupakhsh, Tehran, Iran) for local anesthesia (buccal infiltration for maxillary molars and inferior alveolar nerve block for mandibular molars). The depth of anesthesia was checked twice with electric pulp tester in 15-min intervals. Should two negative responses were elicited *via* a maximum power of electrical impulses (power of 80) the case was condemned as failed anesthesia. For cases with unsuccessful anesthesia the injection was repeated. 

Then the patients were randomly divided into 3 groups (*n*=20). In group 1 the operator pretended to perform a PDL injection with an empty cartridge using a short 20-mm 30-gauge needle set on an intra-ligamental syringe (Anthogyr Manufacturing, Sallanches, France). The injection needle only touched the tissues without any penetration. In group 2, before the onset of treatment 0.2 mL of 2% lidocaine containing 1:80000 epinephrine was injected into PDL at mesiobuccal and distobuccal corners of the tooth with the needle entering the PDL with 45 degrees inclination and the piston was discarded after feeling pressure against injection with the same syringe and needle size. In group 3, PDL injection was performed using 0.2 mL of dexamethasone (8 mg/2 mL, Darupakhsh, Tehran, Iran) with similar conditions and devices. The solutions were prepared by another blinded operator and the treatment process including all injections were done by an independent endodontist. To establish a double blinded design, neither the patient nor the practitioner were aware of the injection solution.

After tooth isolation with rubber-dam and preparation of the access cavity, root canal treatment was done using hand K-files (Dentsply Maillefer, Ballaigues, Switzerland) with step-back technique. The least apical size was set at #30 and apical patency was maintained for all teeth. The canals were irrigated with normal saline and obturated with lateral condensation of gutta-percha and sealer (AH-26, Dentsply, Tulsa Dental, Tulsa, OK, USA). After termination of treatment the access cavity was restored with temporary restoration (Cavit, ESPE-Premier, Norristown, PA, USA) and the occlusion and proximal integrity was meticulously checked to prevent future pain. Before dismissal the patients were briefed about filling of the pain questioner after 6, 12, 24 and 48 h and they were also contacted on the due time. Patients were given a non-numeric VAS ruler which had signs and a similar numerated ruler was kept by the operator who had to correlate the VAS pain signs marked by the patient to the corresponding scores from 0 to 170. The level of pain was scored as follows: *score 0* (mild pain; 0-56), *score 1* (moderate pain; 57-113) and *score 3* (severe pain; 114-170). The patients were also asked to record the type and dosage of analgesics consumption if needed to reduce their post-operative pain.

The data were analyzed using the Kruskal-Wallis and the Chi-square tests. The level of significance was set at 0.05.

## Results

A total of 60 volunteers (30 in each group) met the criteria and were found eligible for this study. The patient distribution into different test groups was not significantly different regarding gender, age, the level of pretreatment pain, the type of tooth and presence/absence of acute apical periodontitis (*P*<0.2) (Table 1). Before treatment the highest and lowest level of post-endodontic pain was found in group 1 (placebo) (106.4±35.4) and group 2 (lidocaine) (97.8±36.07), respectively. However the Kruskal-Wallis test indicated no significant differences (*P*<0.4).

After 6 h the highest and lowest level of post-treatment pain were found in placebo (80±44) and dexamethasone groups (dexamethasone) (35.25±17.47), respectively. The main value of pain significantly decreased in all three groups (*P*<0.05). The amount of pain reduction was 65%, 48% and 25% in dexamethasone, lidocaine and placebo groups, respectively, and this reduction was statistically significant between all groups and also in two-by-two comparison (*P*<0.01). The highest amount of analgesic consumption was in placebo (70%), lidocaine (50%) and dexamethasone (40%) groups, respectively. The Chi-square test did not reveal a significant difference in this regard.

After 12 h, similarly the lowest amount of pain level was found in dexamethasone group while the placebo group had the highest pain (*P*<0.01). Two-by-two comparison revealed that the placebo and lidocaine group and dexamethasone and placebo group were significantly different (*P*<0.01 and *P*<0.001, respectively). There was no report of moderate and severe pain in dexamethasone group. The pattern of taking analgesics followed the similar manner in descending order: placebo (60%), lidocaine (25%) and dexamethasone (25%).

The Chi-square test showed a significant difference between the placebo and other two groups. After 24 h, although the placebo group maintained the highest level of pain perception, the difference was not significant with others (*P*<0.6) and the two-by-two comparison did not show a significant difference between lidocaine and placebo groups, either (*P*<0.6).

**Table 1:** Demographic data of patients in different groups

**Groups **	**Age (y)**	**Gender N (%)**		**Pretreatment pain mean (SD)**	**Tooth N (%)**		**AAP N (%)**	
		**Male**	**Female**		**First molar**	**Second molar**	**Yes**	**No**
**Placebo**	32 (4.6)	9 (45)	11 (55)	106.4 (35.4)	14 (70)	6 (30)	8 (40)	12 (60)
**Lidocaine**	26.1 (9.8)	10 (50)	10 (50)	97.8 (36.07)	11 (55)	9 (45)	8 (40)	12 (60)
**Dexamethasone**	30.35 (4.2)	8 (40)	12 (60)	100.6 (29.61)	14 (70)	6 (30)	12 (60)	8 (40)
***P-*** **value**	0.8	0.7		0.4	0.2		0.2	

**Table 2.** Mean (SD) of pain level in different groups before and after treatment

**Groups **	**Before treatment**	**After 6 h**	**After 12 h**	**After 24 h**
**Placebo**	106.4 (35.4)	80 (44.6)	45 (30.3)	14 (12.8)
**Lidocaine**	97.8 (036.07)	50.45 (26.9)	30.05 (17.06)	11.85 (10.76)
**Dexamethasone**	100.6 (29.61)	35.25 (17.47)	12.3 (35.4)	7.7 (9.91)
***P-*** **value**	0.4	0.05	0.01	0.6

**Table 3:** Analgesic consumption after treatment in different groups

**Groups **	**After 6 h**		**After 12 h**		**After 24 h**		**After 48 h**	
	**Yes**	**No**	**Yes**	**No**	**Yes**	**No**	**Yes**	**No**
**Placebo**	6 (30)	14 (70)	8 (40)	12 (60)	18 (90)	2 (10)	0 (0)	0 (0)
**Lidocaine**	10 (50)	10 (50)	15 (75)	5 (25)	18 (90)	2 (10)	0 (0)	0 (0)
**Dexamethasone**	12 (60)	8 (40)	15 (75)	5 (25)	19 (95)	1 (5)	0 (0)	0 (0)
***P-*** **value**	0.05		0.01		0.6		0.8	

The patients in dexamethasone group maintained the significantly different pain level compared to the placebo (*P*<0.01). The analgesics consumptions were almost similar in all groups without significance.

After 48 h, the highest and lowest pain level were reported in placebo and dexamethasone groups, respectively but the difference was not significant (*P*<0.8). There was no report of moderate and severe pain in any groups after 24 and 48 h. There was no report of analgesic consumption after 48 h (Tables 2 and 3).

## Discussion

This double-blind placebo controlled clinical trial evaluated the effect of intraligamentary injection of dexamethasone and 2% lidocaine on reduction of postoperative pain in patients with irreversible pulpitis using VAS. The VAS score data showed that intraligamentary injection of low dose of dexamethasone was more effective in reduction of post endodontic pain in comparison with lidocaine or placebo group.

Many endodontic patients believe that post-treatment pain is inevitable. The occurrence of a mild postoperative pain is not a rare event even when endodontic treatment has followed all acceptable standards [11]. According to some studies, moderate to severe postoperative pain occurs in only 4-10% of all endodontic patients [12], while some other studies reported higher incidence (almost 50%) [13]. The causative factors of post-treatment pain can be classified as mechanical, chemical, and/or microbial insults to the pulp or periradicular tissues, which are induced or exacerbated during root canal treatment [3]. The intensity of the inflammatory response is directly proportional to the intensity of tissue injury [14]. Since the tissue/vascular events associated with acute inflammation usually result in severe pain, it is conceivable that the greater the intensity of the inflammatory reaction the greater the intensity of potential pain [5, 7]. 

Postoperative pain is usually mild and rarely lasts longer than three days. However, some patients will suffer from a moderate to severe pain that persists for several days even after appropriate endodontic treatment. The persistent pain is often attributed to the release of inflammatory mediators such as prostaglandins, leukotrienes, bradykinin and serotonin. Consequently, peripheral and central hyperalgesia are resulted from the activation and sensitization of nociceptors by these released mediators, especially prostaglandins [15]. 

Various classes of drugs have been studied for the management of post-treatment endodontic pain including NSAID’s and acetaminophen, opioids and steroids [16, 17]. Many studies have proved the effectiveness of preoperative administration of NSAIDS in reducing or suppressing the post-endodontic pain [18, 19]. Clinicians largely rely on non-steroidal anti-inflammatory drugs (NSAIDs) to manage post-endodontic pain [15, 20]. They inhibit the synthesis of prostaglandins through retarding the activity of COX1 and COX2 enzymes, with the first acting in regulation of normal cell activities in stomach, kidneys, endothelial cells, *etc.* and the second appearing in injured and inflamed tissues as an inducible enzyme [21]. Although NSAIDs are remarkably effective in the management of pain and inflammation, their chronic use is limited by a number of adverse effects including gastrointestinal bleeding and ulceration, impaired renal function and inhibition of platelet aggregation due to inhibition of COX1 [17].

The results of the current study showed that PDL injection of dexamethasone significantly reduced the post-endodontic pain levels during the first 12 h postoperatively. Shantiaee *et al.* [22] demonstrated that local infiltration of dexamethasone was more effective than morphine to decrease postoperative endodontic pain during the first 24 h after operation. Dexamethasone is a corticosteroid with strong anti-inflammatory effects 25 times more than that of endogenic cortisol [23]. There are numerous pain-reducing mechanisms of steroids mentioned in the literature. Glucocorticoids affect the immune response by inhibition of cytokine production, specifically interferon γ, granulocyte/monocyte colony stimulating factor (GM-CSF), interleukins 1, 2, 3, 6 (IL-1, IL-2, IL-3, IL-6) and tumor necrosis factor α (TNFα) [23]. Steroids can induce intracellular production of many proteins such as lipocortin that prevents the synthesis of arachidonic acid and thereby reduce the biosynthesis of both cyclooxygenase (COX) and lipoxygenase products, including prostaglandins, leukotrienes and thromboxane related substances [8]. Reduction in pulpal levels of both prostaglandin E2 and IL-8 in irreversibly inflamed pulps after administration of Depomedrol was already reported [24]. Another mechanism of action of steroids can be the reduction of bradykinin mediators through inducing the synthesis of angiotensin converting enzyme (ACE) [23, 25]. Bradykinin activates nociceptors and causes the release of substance P, neurokinin A, and calcitonin gene-related peptide (CGRP) *via* activating the B1 and B2 receptors. The latter receptor mediates the pain in acute inflammation while the pain in chronic inflammation involves an elevation in number of B1 receptors [26]. Reduction of postoperative pain through reducing the bradykinin levels by the administration of glucocorticoids has been demonstrated in many studies [7, 25, 27]. Production of vasocortin that suppresses edema which was not suppressed by NSAIDs, is another important mechanism of pain suppression by steroids [23]. Glucocorticoids may also inhibit neurogenic inflammation by inhibition of the release of neuropeptides [28].

It could therefore be assumed that glucocorticoids have greater anti-inflammatory and analgesic effects than NSAIDs where considering the fact that multiple inflammatory mediators are released or produced during pulpal inflammation [29]. Thus systemic administration of corticosteroid as an alternative strategy to decrease endodontic post-treatment pain might be suggested just in those patients who present with moderate/severe pain with irreversible pulpitis [30]. The point is that almost all of the effects of systemically-administered glucocorticoids do not occur immediately and may only become apparent after several hours or even days after administration [7, 11, 23]. This time is required for changes in gene expression and protein synthesis induced by such medicine. According to Nobuhara *et al. *[31] the average number of PMNs in the apical and middle regions of the PDL space significantly decreased following buccal infiltration of dexamethasone, but not until 48 h postoperatively. However, this study indicated the immediate analgesic effect of dexamethasone after 6 and 12 h which can be due to mechanisms of action other than those including gene expression and protein synthesis. Moreover it is important to note that endodontic treatment *per se* has a major effect on reducing post endodontic pain regardless of analgesic intervention [23].

## Conclusion

The present clinical study represented an effective practical way of intraligamentary injection of a very low-dose dexamethasone to control moderate to severe post-operative pain in patients suffering from symptomatic irreversible pulpitis. Considering the adverse or unwanted effects of systemic administration of NSAIDs or high doses of corticosteroids, local administration of 0.2 mL of dexamethasone may seem a safe alternative way for pain control.

## References

[1] Aggarwal V, Singla M, Rizvi A, Miglani S (2011). Comparative evaluation of local infiltration of articaine, articaine plus ketorolac, and dexamethasone on anesthetic efficacy of inferior alveolar nerve block with lidocaine in patients with irreversible pulpitis. J Endod.

[2] Pochapski MT, Santos FA, de Andrade ED, Sydney GB (2009). Effect of pretreatment dexamethasone on postendodontic pain. Oral Surg Oral Med Oral Pathol Oral Radiol Endod.

[3] Arora M, Sangwan P, Tewari S, Duhan J (2015). Effect of maintaining apical patency on endodontic pain in posterior teeth with pulp necrosis and apical periodontitis: a randomized controlled trial. Int Endod J.

[4] Arias A, de la Macorra JC, Azabal M, Hidalgo JJ, Peters OA (2015). Prospective case controlled clinical study of post-endodontic pain after rotary root canal preparation performed by a single operator. J Dent.

[5] Nixdorf DR, Moana-Filho EJ, Law AS, McGuire LA, Hodges JS, John MT (2010). Frequency of persistent tooth pain after root canal therapy: a systematic review and meta-analysis. J Endod.

[6] Mehrvarzfar P, Shababi B, Sayyad R, Fallahdoost A, Kheradpir K (2008). Effect of supraperiosteal injection of dexamethasone on postoperative pain. Aust Endod J.

[7] Lin S, Levin L, Emodi O, Abu El-Naaj I, Peled M (2006). Etodolac versus dexamethasone effect in reduction of postoperative symptoms following surgical endodontic treatment: a double-blind study. Oral Surg Oral Med Oral Pathol Oral Radiol Endod.

[8] Shahi S, Mokhtari H, Rahimi S, Yavari HR, Narimani S, Abdolrahimi M, Nezafati S (2013). Effect of premedication with ibuprofen and dexamethasone on success rate of inferior alveolar nerve block for teeth with asymptomatic irreversible pulpitis: a randomized clinical trial. J Endod.

[9] Azimi S, Fazlyab M, Sadri D, Saghiri MA, Khosravanifard B, Asgary S (2014). Comparison of pulp response to mineral trioxide aggregate and a bioceramic paste in partial pulpotomy of sound human premolars: a randomized controlled trial. Int Endod J.

[10] Torabinejad M, Cymerman JJ, Frankson M, Lemon RR, Maggio JD, Schilder H (1994). Effectiveness of various medications on postoperative pain following complete instrumentation. J Endod.

[11] Siqueira J, Barnett F (2004). Interappointment pain: mechanisms, diagnosis, and treatment. Endodontic Topics.

[12] Wong AW, Zhang C, Chu CH (2014). A systematic review of nonsurgical single-visit versus multiple-visit endodontic treatment. Clin Cosmet Investig Dent.

[13] DiRenzo A, Gresla T, Johnson BR, Rogers M, Tucker D, BeGole EA (2002). Postoperative pain after 1- and 2-visit root canal therapy. Oral Surg Oral Med Oral Pathol Oral Radiol Endod.

[14] Anjaneyulu K, Nivedhitha MS (2014). Influence of calcium hydroxide on the post-treatment pain in Endodontics: A systematic review. J Conserv Dent.

[15] Arslan H, Topcuoglu HS, Aladag H (2011). Effectiveness of tenoxicam and ibuprofen for pain prevention following endodontic therapy in comparison to placebo: a randomized double-blind clinical trial. J Oral Sci.

[16] Doroschak AM, Bowles WR, Hargreaves KM (1999). Evaluation of the combination of flurbiprofen and tramadol for management of endodontic pain. J Endod.

[17] Rogers MJ, Johnson BR, Remeikis NA, BeGole EA (1999). Comparison of effect of intracanal use of ketorolac tromethamine and dexamethasone with oral ibuprofen on post treatment endodontic pain. J Endod.

[18] Flath RK, Hicks ML, Dionne RA, Pelleu GB (1987). Pain suppression after pulpectomy with preoperative flurbiprofen. J Endod.

[19] Gopikrishna V, Parameswaran A (2003). Effectiveness of prophylactic use of rofecoxib in comparison with ibuprofen on postendodontic pain. J Endod.

[20] Ramazani M, Hamidi MR, Moghaddamnia AA, Ramazani N, Zarenejad N (2013). The Prophylactic Effects of Zintoma and Ibuprofen on Post-endodontic Pain of Molars with Irreversible Pulpitis: A Randomized Clinical Trial. Iran Endod J.

[21] Holt CI, Hutchins MO, Pileggi R (2005). A real time quantitative PCR analysis and correlation of COX-1 and COX-2 enzymes in inflamed dental pulps following administration of three different NSAIDs. J Endod.

[22] Shantiaee Y, Mahjour F, Dianat O (2012). Efficacy comparison of periapical infiltration injection of dexamethasone, morphine and placebo for postoperative endodontic pain. Int Dent J.

[23] Marshall JG (2002). Consideration of steroids for endodontic pain. Endodontic Topics.

[24] Isett J, Reader A, Gallatin E, Beck M, Padgett D (2003). Effect of an intraosseous injection of depo-medrol on pulpal concentrations of PGE2 and IL-8 in untreated irreversible pulpitis. J Endod.

[25] Mohammadi Z (2009). Systemic and local applications of steroids in endodontics: an update review. Int Dent J.

[26] Lepinski AM, Hargreaves KM, Goodis HE, Bowles WR (2000). Bradykinin levels in dental pulp by microdialysis. J Endod.

[27] Tsesis I, Fuss Z, Lin S, Tilinger G, Peled M (2003). Analysis of postoperative symptoms following surgical endodontic treatment. Quintessence Int.

[28] Giuliano C, Smalligan RD, Mitchon G, Chua M (2012). Role of dexamethasone in the prevention of migraine recurrence in the acute care setting: a review. Postgrad Med.

[29] Claffey E, Reader A, Nusstein J, Beck M, Weaver J (2004). Anesthetic efficacy of articaine for inferior alveolar nerve blocks in patients with irreversible pulpitis. J Endod.

[30] Bramy E, Reader A, Gallatin E, Nist R, Beck M, Weaver J (1999). OR 29 Pain reduction in symptomatic, necrotic teeth using an intraosseous injection of Depo-Medrol. J Endod.

[31] Nobuhara WK, Carnes DL, Gilles JA (1993). Anti-inflammatory effects of dexamethasone on periapical tissues following endodontic overinstrumentation. J Endod.

